# *catena*-Poly[[(2-amino-1,3-benzothia­zole-6-carboxyl­ato-κ^2^ *O*,*O*′)(2,2′-bipyridyl-κ^2^ *N*,*N*′)cadmium]-μ-2-amino-1,3-benzothia­zole-6-carboxyl­ato-κ^3^ *N* ^1^:*O*,*O*′]

**DOI:** 10.1107/S160053681201642X

**Published:** 2012-04-21

**Authors:** Dan Gao, Xin Fang, Ke-Ke Zhang, Li-Mao Cai, Jun-Dong Wang

**Affiliations:** a Department of Chemistry, University of Fuzhou, Fuzhou350108, People’s Republic of China

## Abstract

In the title coordination polymer, [Cd(C_8_H_5_N_2_O_2_S)_2_(C_10_H_8_N_2_)]_*n*_, the Cd^II^ ion is coordinated by a bidentate 2,2-bipyridyl ligand, two *O*,*O*′-chelating 2-amino-1,3-benzothia­zole-6-carboxyl­ate (ABTC) ligands and one *N*-bonded ABTC ligand. The resulting CdN_3_O_4_ coordination polyhedron approximates to a very distorted penta­gonal bipramid with one O and one N atom in axial positions. One of the ABTC ligands is bridging to an adjacent metal atom, generating an infinite chain propagating in [100]. A three-dimensional network is constructed from N—H⋯O and N—H⋯N hydrogen bonds and aromatic π–π stacking inter­actions [centroid–centroid separations = 3.641 (2) and 3.682 (3) Å].

## Related literature

For our recent work on the design and sythesis of benzothia­zole coordination networks, see: Fang *et al.* (2010 ▶); Lei *et al.* (2010 ▶). For the synthesis of the ligand, see: Das *et al.* (2003 ▶).
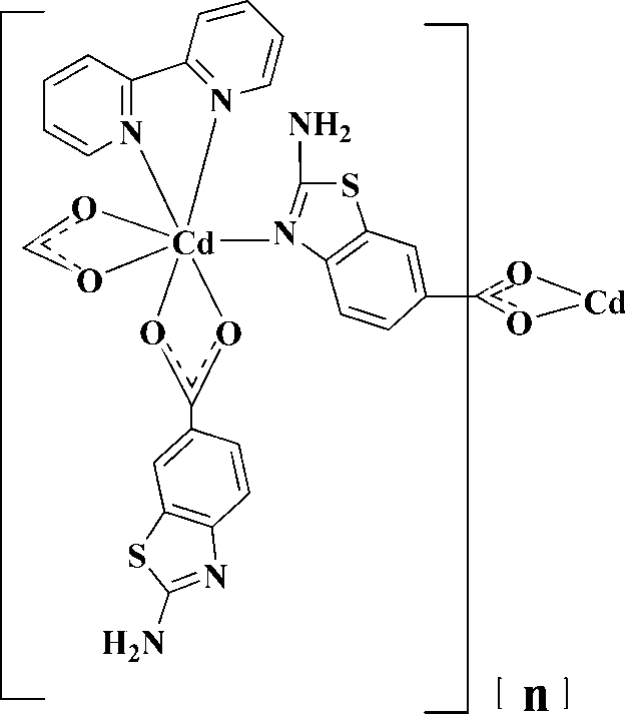


## Experimental

### 

#### Crystal data

[Cd(C_8_H_5_N_2_O_2_S)_2_(C_10_H_8_N_2_)]
*M* *_r_* = 640.87
Triclinic, 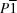

*a* = 9.977 (2) Å
*b* = 11.715 (2) Å
*c* = 11.734 (2) Å
α = 65.28 (3)°
β = 77.52 (3)°
γ = 77.15 (3)°
*V* = 1202.8 (4) Å^3^
*Z* = 2
Mo *K*α radiation
μ = 1.13 mm^−1^
*T* = 173 K
0.27 × 0.18 × 0.13 mm

#### Data collection

Rigaku Saturn 724 CCD area-detector diffractometer
Absorption correction: numerical (*NUMABS*; Higashi, 2000 ▶) *T*_min_ = 0.837, *T*_max_ = 1.000
10158 measured reflections
5296 independent reflections
5015 reflections with *I* > 2σ(*I*)
*R*_int_ = 0.037

#### Refinement

*R*[*F* ^2^ > 2σ(*F* ^2^)] = 0.040
*wR*(*F* ^2^) = 0.088
*S* = 1.08
5296 reflections
352 parameters
H-atom parameters constrained
Δρ_max_ = 1.50 e Å^−3^
Δρ_min_ = −0.72 e Å^−3^

### 

Data collection: *CrystalClear* (Rigaku, 2007 ▶); cell refinement: *CrystalClear*; data reduction: *CrystalClear*; program(s) used to solve structure: *SHELXS97* (Sheldrick, 2008 ▶); program(s) used to refine structure: *SHELXL97* (Sheldrick, 2008 ▶); molecular graphics: *ORTEX* (McArdle, 1995 ▶); software used to prepare material for publication: *SHELXL97*.

## Supplementary Material

Crystal structure: contains datablock(s) I, global. DOI: 10.1107/S160053681201642X/hb6715sup1.cif

Structure factors: contains datablock(s) I. DOI: 10.1107/S160053681201642X/hb6715Isup2.hkl

Additional supplementary materials:  crystallographic information; 3D view; checkCIF report

## Figures and Tables

**Table 1:** Selected bond lengths (Å)

Cd1—N3	2.345 (3)
Cd1—O3^i^	2.372 (2)
Cd1—O1	2.381 (3)
Cd1—N1	2.391 (3)
Cd1—O2	2.415 (3)
Cd1—O4^i^	2.422 (2)
Cd1—N2	2.484 (3)

Symmetry code: (i) 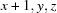.

**Table 2:** Hydrogen-bond geometry (Å, °)

*D*—H⋯*A*	*D*—H	H⋯*A*	*D*⋯*A*	*D*—H⋯*A*
N6—H6*A*⋯O3^ii^	0.88	2.10	2.917 (4)	155
N6—H6*B*⋯O3^iii^	0.88	2.12	2.996 (4)	173
N4—H4*A*⋯O4^i^	0.88	2.25	3.101 (4)	162
N4—H4*B*⋯N5^iv^	0.88	2.21	3.066 (4)	163

Symmetry codes: (i) 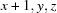; (ii) 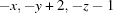; (iii) 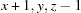; (iv) 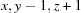.

**Fig. 1.**
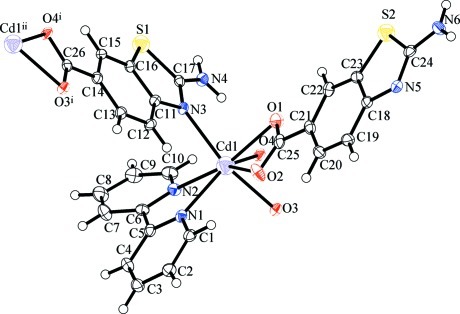
The coordination environment of Cd atom, drawn with 50% probability displacement ellipsoids. [Symmetry codes: (i)1 + x,y,z; (ii)-1 + x,y,z]

**Fig. 2.**
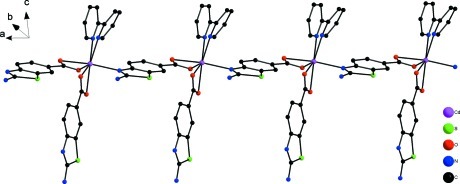
A Chain formed by Cd1—N3 bond. H atoms are omitted.

**Fig. 3.**
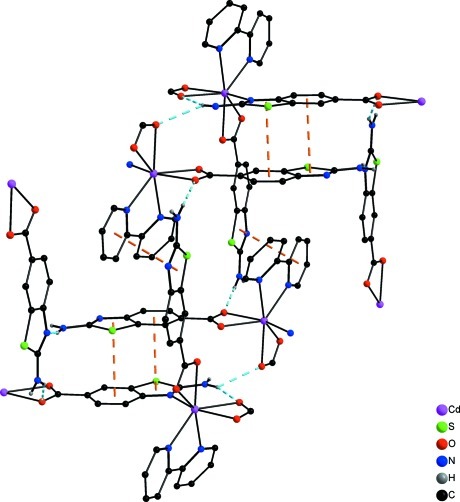
Three-dimensional net with hydrogen bonds and π-π interactions. H atoms are omitted except those forming hydrogen bonds.

## supplementary crystallographic information

### Comment

As part of our ongoing studies of benzothiaole-based coordination networks (Fang *et al.*, 2010; Lei *et al.*, 2010), we now report the structure of a coordination polymer of cadmium and 2-amino-1,3-benzothiazole-6-carboxylate (ABTC) with 2,2'-bipyridine (bpy) as second ligand.

The polymer is a triclinic system crystal and it crystallizes in the space group of P-1. In the asymmetric unit (Fig. 1), center Cd (II) is seven-coordinated with (*κ*1, *κ*2)-*µ*2 coordination model, where one bpy provides two N atoms, one ABTC affords two O atoms, and the another ABTC affords two O atoms and one N atom of thiazole. The four Cd—O bonds are Cd1—O1, Cd1—O2, Cd1—O3, and Cd1—O4, with distance of 2.380 (7) Å, 2.415 (7) Å, 2.371 (3) Å, and 2.422 (6) Å, respectively. The three Cd—N bonds are Cd1—N1, Cd1—N2, and Cd1—N3, with distance of 2.390 (8) Å, 2.484 (1) Å, and 2.345 (6) Å, respectively, where the Cd1—N2 distance is slightly longer than common distance of Cd and N.

The asymmetric units are forming chains extending along the *a* axis (Fig. 2), through the one ABTC coordinated to two Cd(II) simultaneously by O from carboxylate and N from thiazole ring, respectively. Furthermore, three-dimensional supermolecular net (Fig. 3) are constructed by hydrogen bonds, as listed in Table 1, and π-π interactions [between thiazole and benzene rings (with certroid-centroid distance of 3.682 (3) Å and centroid-ring plane distance of 3.350 (9) and 3.404 (3) Å), and between the thiazole and bpy rings (with certroid-centroid distance of 3.641 (2) Å and centroid-ring plane distance of 3.478 (9) and 3.331 (7) Å)].

### Experimental

The 2-aminobenzothiazole-6-carboxylic acid ligand was obtained by hydrolyzing of ethyl-2-amion-1,3-benzothiazole-6-carboxylate (Das *et al.* 2003). The mixture of cadmium carbonate (0.0172 g, 0.1 mol), 2-aminobenzothiazole-6-carboxylic acid (0.0388 g, 0.2 mol), 2,2'-bipyridine (0.0160 g, 0.1 mol) and H_2_O (8 ml) was sealed in a 23 ml stainless-steel reactor with Teflon liner and heated (283 K per hour) from room temperature to 423 K and kept at 423 K for 4 days, then cooled (279 K per hour) to room temperature. Colorless prisms were obtained.

### Refinement

All hydrogen atoms were positioned geometrically and refined in a riding model approximation with *U*_iso_ (H) = 1.2 *U*_eq_ (C) or *U*_eq_ (N).

### Crystal data

**Table:**

[Cd(C_8_H_5_N_2_O_2_S)_2_(C_10_H_8_N_2_)]	*Z* = 2
*M**_r_* = 640.87	*F*(000) = 656.0
Triclinic, *P*1	*D*_x_ = 1.770 Mg m^−^^3^
Hall symbol: -P 1	Mo *K*α radiation, λ = 0.71073 Å
*a* = 9.977 (2) Å	Cell parameters from 4682 reflections
*b* = 11.715 (2) Å	θ = 3.1–27.6°
*c* = 11.734 (2) Å	µ = 1.13 mm^−^^1^
α = 65.28 (3)°	*T* = 173 K
β = 77.52 (3)°	Prism, colourless
γ = 77.15 (3)°	0.27 × 0.18 × 0.13 mm
*V* = 1202.8 (4) Å^3^	

### Data collection

**Table:**

Rigaku Saturn 724 CCD area-detector diffractometer	5296 independent reflections
Radiation source: fine-focus sealed tube	5015 reflections with *I* > 2σ(*I*)
Graphite monochromator	*R*_int_ = 0.037
Detector resolution: 28.5714 pixels mm^-1^	θ_max_ = 27.6°, θ_min_ = 3.6°
dtprofit.ref scans	*h* = −12→12
Absorption correction: numerical (*NUMABS*; Higashi, 2000)	*k* = −14→15
*T*_min_ = 0.837, *T*_max_ = 1.000	*l* = −14→15
10158 measured reflections	

### Refinement

**Table:**

Refinement on *F*^2^	Primary atom site location: structure-invariant direct methods
Least-squares matrix: full	Secondary atom site location: difference Fourier map
*R*[*F*^2^ > 2σ(*F*^2^)] = 0.040	Hydrogen site location: inferred from neighbouring sites
*wR*(*F*^2^) = 0.088	H-atom parameters constrained
*S* = 1.08	*w* = 1/[σ^2^(*F*_o_^2^) + (0.0263*P*)^2^ + 2.5814*P*] where *P* = (*F*_o_^2^ + 2*F*_c_^2^)/3
5296 reflections	(Δ/σ)_max_ = 0.001
352 parameters	Δρ_max_ = 1.50 e Å^−^^3^
0 restraints	Δρ_min_ = −0.72 e Å^−^^3^

### Special details

**Table:**

Geometry. All e.s.d.'s (except the e.s.d. in the dihedral angle between two l.s. planes) are estimated using the full covariance matrix. The cell e.s.d.'s are taken into account individually in the estimation of e.s.d.'s in distances, angles and torsion angles; correlations between e.s.d.'s in cell parameters are only used when they are defined by crystal symmetry. An approximate (isotropic) treatment of cell e.s.d.'s is used for estimating e.s.d.'s involving l.s. planes.
Refinement. Refinement of *F*^2^ against ALL reflections. The weighted *R*-factor *wR* and goodness of fit *S* are based on *F*^2^, conventional *R*-factors *R* are based on *F*, with *F* set to zero for negative *F*^2^. The threshold expression of *F*^2^ > σ(*F*^2^) is used only for calculating *R*-factors(gt) *etc*. and is not relevant to the choice of reflections for refinement. *R*-factors based on *F*^2^ are statistically about twice as large as those based on *F*, and *R*- factors based on ALL data will be even larger.

### Fractional atomic coordinates and isotropic or equivalent isotropic displacement parameters (Å^2^)

**Table:**

	*x*	*y*	*z*	*U*_iso_*/*U*_eq_	
Cd1	0.30365 (2)	0.70429 (2)	0.09531 (2)	0.01412 (8)	
S2	0.41833 (9)	0.83130 (8)	−0.62591 (8)	0.01725 (17)	
O1	0.3596 (3)	0.7157 (2)	−0.1166 (2)	0.0248 (6)	
O2	0.2554 (3)	0.8927 (3)	−0.0912 (2)	0.0271 (6)	
N1	0.1528 (3)	0.8327 (3)	0.1983 (3)	0.0155 (6)	
N2	0.2833 (3)	0.5972 (3)	0.3298 (3)	0.0180 (6)	
N5	0.3521 (3)	1.0786 (3)	−0.6983 (3)	0.0158 (6)	
N6	0.4267 (3)	1.0043 (3)	−0.8634 (3)	0.0213 (6)	
H6A	0.4161	1.0813	−0.9227	0.026*	
H6B	0.4567	0.9382	−0.8844	0.026*	
S1	0.06488 (8)	0.34375 (7)	0.19139 (8)	0.01547 (16)	
N3	0.1635 (3)	0.5537 (3)	0.1288 (3)	0.0140 (5)	
N4	0.3306 (3)	0.3686 (3)	0.1717 (3)	0.0178 (6)	
H4A	0.3994	0.4110	0.1563	0.021*	
H4B	0.3471	0.2856	0.1940	0.021*	
O4	−0.4784 (2)	0.5623 (2)	0.1301 (2)	0.0175 (5)	
O3	−0.4823 (2)	0.7664 (2)	0.0858 (2)	0.0172 (5)	
C1	0.1130 (3)	0.9574 (3)	0.1355 (3)	0.0179 (7)	
H1	0.1300	0.9911	0.0456	0.021*	
C2	0.0484 (4)	1.0395 (3)	0.1951 (3)	0.0196 (7)	
H2	0.0224	1.1273	0.1470	0.023*	
C3	0.0224 (4)	0.9910 (3)	0.3263 (3)	0.0201 (7)	
H3	−0.0199	1.0452	0.3699	0.024*	
C4	0.0592 (3)	0.8621 (3)	0.3925 (3)	0.0195 (7)	
H4	0.0400	0.8262	0.4824	0.023*	
C5	0.1251 (3)	0.7848 (3)	0.3261 (3)	0.0160 (6)	
C6	0.1758 (4)	0.6489 (3)	0.3931 (3)	0.0171 (7)	
C7	0.1184 (4)	0.5801 (4)	0.5154 (3)	0.0245 (8)	
H7	0.0404	0.6179	0.5569	0.029*	
C8	0.1773 (4)	0.4542 (4)	0.5763 (3)	0.0267 (8)	
H8	0.1392	0.4042	0.6596	0.032*	
C9	0.2915 (4)	0.4038 (3)	0.5134 (3)	0.0249 (8)	
H9	0.3361	0.3195	0.5541	0.030*	
C10	0.3402 (4)	0.4771 (3)	0.3908 (4)	0.0236 (7)	
H10	0.4178	0.4408	0.3475	0.028*	
C11	0.0212 (3)	0.5869 (3)	0.1247 (3)	0.0144 (6)	
C12	−0.0528 (3)	0.7099 (3)	0.0958 (3)	0.0155 (6)	
H12	−0.0063	0.7795	0.0771	0.019*	
C13	−0.1952 (3)	0.7287 (3)	0.0949 (3)	0.0163 (6)	
H13	−0.2461	0.8115	0.0777	0.020*	
C14	−0.2650 (3)	0.6284 (3)	0.1187 (3)	0.0150 (6)	
C15	−0.1930 (3)	0.5050 (3)	0.1483 (3)	0.0147 (6)	
H15	−0.2395	0.4360	0.1651	0.018*	
C16	−0.0512 (3)	0.4864 (3)	0.1522 (3)	0.0135 (6)	
C17	0.2006 (3)	0.4302 (3)	0.1613 (3)	0.0139 (6)	
C18	0.3359 (3)	1.0268 (3)	−0.5664 (3)	0.0163 (6)	
C19	0.2953 (4)	1.0964 (3)	−0.4897 (3)	0.0193 (7)	
H19	0.2735	1.1864	−0.5264	0.023*	
C20	0.2872 (3)	1.0324 (3)	−0.3587 (3)	0.0191 (7)	
H20	0.2600	1.0796	−0.3064	0.023*	
C21	0.3182 (3)	0.9002 (3)	−0.3027 (3)	0.0176 (7)	
C22	0.3572 (3)	0.8301 (3)	−0.3795 (3)	0.0183 (7)	
H22	0.3774	0.7400	−0.3426	0.022*	
C23	0.3659 (3)	0.8933 (3)	−0.5094 (3)	0.0161 (6)	
C24	0.3974 (3)	0.9876 (3)	−0.7413 (3)	0.0172 (7)	
C25	0.3105 (3)	0.8324 (3)	−0.1607 (3)	0.0187 (7)	
C26	−0.4178 (3)	0.6531 (3)	0.1118 (3)	0.0136 (6)	

### Atomic displacement parameters (Å^2^)

**Table:**

	*U*^11^	*U*^22^	*U*^33^	*U*^12^	*U*^13^	*U*^23^
Cd1	0.01171 (12)	0.01595 (13)	0.01451 (12)	−0.00318 (9)	−0.00112 (8)	−0.00551 (9)
S2	0.0197 (4)	0.0161 (4)	0.0165 (4)	−0.0033 (3)	−0.0023 (3)	−0.0066 (3)
O1	0.0318 (15)	0.0235 (13)	0.0170 (12)	−0.0069 (11)	−0.0032 (11)	−0.0045 (10)
O2	0.0271 (14)	0.0345 (15)	0.0163 (12)	0.0047 (12)	−0.0040 (11)	−0.0107 (11)
N1	0.0119 (13)	0.0193 (14)	0.0155 (13)	−0.0037 (11)	−0.0031 (11)	−0.0057 (11)
N2	0.0215 (15)	0.0161 (14)	0.0160 (13)	−0.0020 (11)	−0.0040 (11)	−0.0057 (11)
N5	0.0151 (14)	0.0150 (13)	0.0162 (13)	−0.0039 (11)	−0.0023 (11)	−0.0042 (11)
N6	0.0279 (16)	0.0182 (14)	0.0164 (14)	−0.0030 (12)	0.0008 (12)	−0.0075 (12)
S1	0.0135 (4)	0.0134 (4)	0.0192 (4)	−0.0021 (3)	−0.0022 (3)	−0.0061 (3)
N3	0.0088 (13)	0.0160 (13)	0.0164 (13)	−0.0019 (10)	−0.0019 (10)	−0.0055 (11)
N4	0.0134 (13)	0.0166 (14)	0.0221 (14)	−0.0005 (11)	−0.0021 (11)	−0.0074 (11)
O4	0.0112 (11)	0.0196 (12)	0.0232 (12)	−0.0049 (9)	−0.0019 (9)	−0.0086 (10)
O3	0.0142 (11)	0.0171 (11)	0.0198 (12)	−0.0035 (9)	−0.0024 (9)	−0.0061 (9)
C1	0.0201 (17)	0.0195 (16)	0.0151 (15)	−0.0069 (14)	−0.0032 (13)	−0.0053 (13)
C2	0.0195 (17)	0.0151 (16)	0.0229 (17)	−0.0007 (13)	−0.0034 (14)	−0.0069 (13)
C3	0.0178 (17)	0.0219 (17)	0.0222 (17)	−0.0016 (14)	−0.0015 (14)	−0.0113 (14)
C4	0.0172 (17)	0.0248 (18)	0.0160 (16)	−0.0001 (14)	−0.0013 (13)	−0.0094 (14)
C5	0.0128 (15)	0.0195 (16)	0.0155 (15)	−0.0036 (13)	−0.0018 (12)	−0.0061 (13)
C6	0.0182 (17)	0.0178 (16)	0.0170 (16)	−0.0017 (13)	−0.0053 (13)	−0.0076 (13)
C7	0.028 (2)	0.0244 (18)	0.0159 (16)	0.0004 (15)	−0.0001 (15)	−0.0068 (14)
C8	0.038 (2)	0.0246 (19)	0.0149 (16)	−0.0051 (16)	−0.0040 (15)	−0.0046 (14)
C9	0.033 (2)	0.0175 (17)	0.0215 (18)	0.0008 (15)	−0.0111 (16)	−0.0038 (14)
C10	0.0255 (19)	0.0206 (17)	0.0261 (18)	0.0006 (15)	−0.0077 (15)	−0.0105 (15)
C11	0.0148 (16)	0.0154 (15)	0.0126 (14)	−0.0040 (12)	−0.0028 (12)	−0.0038 (12)
C12	0.0189 (17)	0.0134 (15)	0.0138 (15)	−0.0058 (13)	−0.0024 (12)	−0.0034 (12)
C13	0.0179 (17)	0.0167 (16)	0.0132 (15)	−0.0043 (13)	−0.0015 (12)	−0.0042 (12)
C14	0.0142 (16)	0.0186 (16)	0.0120 (14)	−0.0039 (13)	−0.0017 (12)	−0.0054 (12)
C15	0.0176 (16)	0.0155 (15)	0.0129 (14)	−0.0064 (13)	0.0005 (12)	−0.0066 (12)
C16	0.0139 (15)	0.0133 (15)	0.0117 (14)	−0.0019 (12)	−0.0014 (12)	−0.0034 (12)
C17	0.0119 (15)	0.0186 (16)	0.0116 (14)	−0.0017 (12)	−0.0017 (12)	−0.0065 (12)
C18	0.0146 (16)	0.0208 (17)	0.0151 (15)	−0.0058 (13)	−0.0010 (12)	−0.0075 (13)
C19	0.0180 (17)	0.0204 (17)	0.0215 (17)	−0.0031 (14)	−0.0025 (14)	−0.0101 (14)
C20	0.0171 (17)	0.0240 (18)	0.0190 (16)	−0.0027 (14)	−0.0013 (13)	−0.0118 (14)
C21	0.0128 (16)	0.0245 (17)	0.0153 (16)	−0.0021 (13)	−0.0012 (13)	−0.0083 (13)
C22	0.0168 (17)	0.0170 (16)	0.0164 (16)	−0.0034 (13)	−0.0006 (13)	−0.0024 (13)
C23	0.0160 (16)	0.0172 (16)	0.0159 (15)	−0.0028 (13)	−0.0026 (13)	−0.0066 (13)
C24	0.0157 (16)	0.0204 (17)	0.0177 (16)	−0.0067 (13)	−0.0042 (13)	−0.0066 (13)
C25	0.0097 (15)	0.0285 (19)	0.0176 (16)	−0.0045 (14)	−0.0020 (13)	−0.0077 (14)
C26	0.0128 (15)	0.0168 (15)	0.0104 (14)	−0.0022 (12)	−0.0010 (12)	−0.0046 (12)

### Geometric parameters (Å, º)

**Table:**

Cd1—N3	2.345 (3)	C3—C4	1.384 (5)
Cd1—O3^i^	2.372 (2)	C3—H3	0.9500
Cd1—O1	2.381 (3)	C4—C5	1.401 (5)
Cd1—N1	2.391 (3)	C4—H4	0.9500
Cd1—O2	2.415 (3)	C5—C6	1.474 (5)
Cd1—O4^i^	2.422 (2)	C6—C7	1.386 (5)
Cd1—N2	2.484 (3)	C7—C8	1.395 (5)
S2—C23	1.739 (3)	C7—H7	0.9500
S2—C24	1.763 (4)	C8—C9	1.376 (5)
O1—C25	1.264 (4)	C8—H8	0.9500
O2—C25	1.258 (4)	C9—C10	1.376 (5)
N1—C1	1.342 (4)	C9—H9	0.9500
N1—C5	1.352 (4)	C10—H10	0.9500
N2—C10	1.339 (4)	C11—C12	1.402 (5)
N2—C6	1.346 (4)	C11—C16	1.404 (4)
N5—C24	1.316 (4)	C12—C13	1.390 (5)
N5—C18	1.392 (4)	C12—H12	0.9500
N6—C24	1.337 (4)	C13—C14	1.396 (4)
N6—H6A	0.8800	C13—H13	0.9500
N6—H6B	0.8800	C14—C15	1.397 (5)
S1—C16	1.753 (3)	C14—C26	1.500 (4)
S1—C17	1.758 (3)	C15—C16	1.390 (4)
N3—C17	1.318 (4)	C15—H15	0.9500
N3—C11	1.392 (4)	C18—C19	1.396 (5)
N4—C17	1.340 (4)	C18—C23	1.410 (5)
N4—H4A	0.8800	C19—C20	1.393 (5)
N4—H4B	0.8800	C19—H19	0.9500
O4—C26	1.258 (4)	C20—C21	1.396 (5)
O4—Cd1^ii^	2.422 (2)	C20—H20	0.9500
O3—C26	1.279 (4)	C21—C22	1.401 (5)
O3—Cd1^ii^	2.372 (2)	C21—C25	1.509 (5)
C1—C2	1.387 (5)	C22—C23	1.380 (5)
C1—H1	0.9500	C22—H22	0.9500
C2—C3	1.386 (5)	C26—Cd1^ii^	2.742 (3)
C2—H2	0.9500		
			
N3—Cd1—O3^i^	153.13 (9)	N1—C5—C6	116.9 (3)
N3—Cd1—O1	85.88 (10)	C4—C5—C6	121.3 (3)
O3^i^—Cd1—O1	92.07 (9)	N2—C6—C7	122.2 (3)
N3—Cd1—N1	101.19 (9)	N2—C6—C5	116.1 (3)
O3^i^—Cd1—N1	99.05 (9)	C7—C6—C5	121.7 (3)
O1—Cd1—N1	135.28 (9)	C6—C7—C8	118.7 (3)
N3—Cd1—O2	110.44 (10)	C6—C7—H7	120.6
O3^i^—Cd1—O2	89.76 (9)	C8—C7—H7	120.6
O1—Cd1—O2	54.93 (9)	C9—C8—C7	118.7 (3)
N1—Cd1—O2	81.78 (9)	C9—C8—H8	120.6
N3—Cd1—O4^i^	98.05 (9)	C7—C8—H8	120.6
O3^i^—Cd1—O4^i^	55.09 (8)	C8—C9—C10	119.1 (3)
O1—Cd1—O4^i^	85.33 (9)	C8—C9—H9	120.4
N1—Cd1—O4^i^	135.73 (9)	C10—C9—H9	120.4
O2—Cd1—O4^i^	127.19 (9)	N2—C10—C9	123.0 (3)
N3—Cd1—N2	80.49 (10)	N2—C10—H10	118.5
O3^i^—Cd1—N2	91.08 (9)	C9—C10—H10	118.5
O1—Cd1—N2	155.59 (9)	N3—C11—C12	125.5 (3)
N1—Cd1—N2	67.79 (10)	N3—C11—C16	115.7 (3)
O2—Cd1—N2	149.31 (9)	C12—C11—C16	118.8 (3)
O4^i^—Cd1—N2	76.72 (9)	C13—C12—C11	119.0 (3)
N3—Cd1—C25	99.34 (10)	C13—C12—H12	120.5
O3^i^—Cd1—C25	90.67 (9)	C11—C12—H12	120.5
O1—Cd1—C25	27.53 (9)	C12—C13—C14	121.5 (3)
N1—Cd1—C25	108.67 (10)	C12—C13—H13	119.3
O2—Cd1—C25	27.41 (10)	C14—C13—H13	119.3
O4^i^—Cd1—C25	106.96 (10)	C13—C14—C15	120.2 (3)
N2—Cd1—C25	176.27 (10)	C13—C14—C26	120.0 (3)
N3—Cd1—C26^i^	125.36 (10)	C15—C14—C26	119.8 (3)
O3^i^—Cd1—C26^i^	27.78 (9)	C16—C15—C14	118.0 (3)
O1—Cd1—C26^i^	88.58 (10)	C16—C15—H15	121.0
N1—Cd1—C26^i^	119.61 (9)	C14—C15—H15	121.0
O2—Cd1—C26^i^	110.00 (10)	C15—C16—C11	122.4 (3)
O4^i^—Cd1—C26^i^	27.31 (8)	C15—C16—S1	128.6 (2)
N2—Cd1—C26^i^	83.04 (10)	C11—C16—S1	109.0 (2)
C25—Cd1—C26^i^	99.99 (10)	N3—C17—N4	125.4 (3)
C23—S2—C24	88.73 (16)	N3—C17—S1	115.4 (2)
C25—O1—Cd1	92.0 (2)	N4—C17—S1	119.2 (2)
C25—O2—Cd1	90.5 (2)	N5—C18—C19	125.3 (3)
C1—N1—C5	118.0 (3)	N5—C18—C23	115.5 (3)
C1—N1—Cd1	121.9 (2)	C19—C18—C23	119.2 (3)
C5—N1—Cd1	119.2 (2)	C20—C19—C18	119.2 (3)
C10—N2—C6	118.1 (3)	C20—C19—H19	120.4
C10—N2—Cd1	123.5 (2)	C18—C19—H19	120.4
C6—N2—Cd1	114.9 (2)	C19—C20—C21	121.4 (3)
C24—N5—C18	109.9 (3)	C19—C20—H20	119.3
C24—N6—H6A	120.0	C21—C20—H20	119.3
C24—N6—H6B	120.0	C20—C21—C22	119.5 (3)
H6A—N6—H6B	120.0	C20—C21—C25	120.7 (3)
C16—S1—C17	89.23 (15)	C22—C21—C25	119.8 (3)
C17—N3—C11	110.7 (3)	C23—C22—C21	119.3 (3)
C17—N3—Cd1	127.6 (2)	C23—C22—H22	120.4
C11—N3—Cd1	121.6 (2)	C21—C22—H22	120.4
C17—N4—H4A	120.0	C22—C23—C18	121.4 (3)
C17—N4—H4B	120.0	C22—C23—S2	128.8 (3)
H4A—N4—H4B	120.0	C18—C23—S2	109.7 (2)
C26—O4—Cd1^ii^	90.62 (19)	N5—C24—N6	125.6 (3)
C26—O3—Cd1^ii^	92.41 (19)	N5—C24—S2	116.1 (3)
N1—C1—C2	123.4 (3)	N6—C24—S2	118.3 (3)
N1—C1—H1	118.3	O2—C25—O1	122.6 (3)
C2—C1—H1	118.3	O2—C25—C21	119.5 (3)
C3—C2—C1	118.6 (3)	O1—C25—C21	117.9 (3)
C3—C2—H2	120.7	O2—C25—Cd1	62.11 (18)
C1—C2—H2	120.7	O1—C25—Cd1	60.52 (18)
C4—C3—C2	118.8 (3)	C21—C25—Cd1	178.2 (2)
C4—C3—H3	120.6	O4—C26—O3	121.9 (3)
C2—C3—H3	120.6	O4—C26—C14	119.2 (3)
C3—C4—C5	119.5 (3)	O3—C26—C14	118.9 (3)
C3—C4—H4	120.3	O4—C26—Cd1^ii^	62.07 (17)
C5—C4—H4	120.3	O3—C26—Cd1^ii^	59.82 (16)
N1—C5—C4	121.7 (3)	C14—C26—Cd1^ii^	178.6 (2)
			
N3—Cd1—O1—C25	119.4 (2)	C17—N3—C11—C12	178.7 (3)
O3^i^—Cd1—O1—C25	−87.5 (2)	Cd1—N3—C11—C12	2.8 (4)
N1—Cd1—O1—C25	17.6 (3)	C17—N3—C11—C16	−1.3 (4)
O2—Cd1—O1—C25	0.78 (19)	Cd1—N3—C11—C16	−177.1 (2)
O4^i^—Cd1—O1—C25	−142.2 (2)	N3—C11—C12—C13	179.8 (3)
N2—Cd1—O1—C25	175.3 (2)	C16—C11—C12—C13	−0.3 (5)
C26^i^—Cd1—O1—C25	−115.0 (2)	C11—C12—C13—C14	−1.7 (5)
N3—Cd1—O2—C25	−70.0 (2)	C12—C13—C14—C15	2.1 (5)
O3^i^—Cd1—O2—C25	91.9 (2)	C12—C13—C14—C26	−177.5 (3)
O1—Cd1—O2—C25	−0.78 (19)	C13—C14—C15—C16	−0.4 (5)
N1—Cd1—O2—C25	−168.9 (2)	C26—C14—C15—C16	179.3 (3)
O4^i^—Cd1—O2—C25	48.1 (2)	C14—C15—C16—C11	−1.7 (5)
N2—Cd1—O2—C25	−176.38 (19)	C14—C15—C16—S1	177.6 (2)
C26^i^—Cd1—O2—C25	72.5 (2)	N3—C11—C16—C15	−178.1 (3)
N3—Cd1—N1—C1	−120.1 (2)	C12—C11—C16—C15	2.0 (5)
O3^i^—Cd1—N1—C1	77.7 (2)	N3—C11—C16—S1	2.6 (3)
O1—Cd1—N1—C1	−24.6 (3)	C12—C11—C16—S1	−177.4 (2)
O2—Cd1—N1—C1	−10.7 (2)	C17—S1—C16—C15	178.3 (3)
O4^i^—Cd1—N1—C1	125.9 (2)	C17—S1—C16—C11	−2.4 (2)
N2—Cd1—N1—C1	165.2 (3)	C11—N3—C17—N4	178.8 (3)
C25—Cd1—N1—C1	−16.1 (3)	Cd1—N3—C17—N4	−5.6 (5)
C26^i^—Cd1—N1—C1	97.6 (3)	C11—N3—C17—S1	−0.7 (3)
N3—Cd1—N1—C5	71.2 (2)	Cd1—N3—C17—S1	174.85 (14)
O3^i^—Cd1—N1—C5	−91.0 (2)	C16—S1—C17—N3	1.8 (3)
O1—Cd1—N1—C5	166.7 (2)	C16—S1—C17—N4	−177.7 (3)
O2—Cd1—N1—C5	−179.5 (2)	C24—N5—C18—C19	177.1 (3)
O4^i^—Cd1—N1—C5	−42.9 (3)	C24—N5—C18—C23	−1.9 (4)
N2—Cd1—N1—C5	−3.6 (2)	N5—C18—C19—C20	−178.1 (3)
C25—Cd1—N1—C5	175.2 (2)	C23—C18—C19—C20	0.9 (5)
C26^i^—Cd1—N1—C5	−71.1 (3)	C18—C19—C20—C21	−0.3 (5)
N3—Cd1—N2—C10	67.9 (3)	C19—C20—C21—C22	−0.6 (5)
O3^i^—Cd1—N2—C10	−86.4 (3)	C19—C20—C21—C25	179.3 (3)
O1—Cd1—N2—C10	11.0 (4)	C20—C21—C22—C23	0.8 (5)
N1—Cd1—N2—C10	174.2 (3)	C25—C21—C22—C23	−179.0 (3)
O2—Cd1—N2—C10	−177.8 (2)	C21—C22—C23—C18	−0.2 (5)
O4^i^—Cd1—N2—C10	−32.8 (3)	C21—C22—C23—S2	176.9 (3)
C25—Cd1—N2—C10	155.6 (14)	N5—C18—C23—C22	178.5 (3)
C26^i^—Cd1—N2—C10	−59.8 (3)	C19—C18—C23—C22	−0.6 (5)
N3—Cd1—N2—C6	−90.6 (2)	N5—C18—C23—S2	0.8 (4)
O3^i^—Cd1—N2—C6	115.1 (2)	C19—C18—C23—S2	−178.3 (3)
O1—Cd1—N2—C6	−147.5 (2)	C24—S2—C23—C22	−177.1 (3)
N1—Cd1—N2—C6	15.7 (2)	C24—S2—C23—C18	0.3 (3)
O2—Cd1—N2—C6	23.7 (3)	C18—N5—C24—N6	−178.6 (3)
O4^i^—Cd1—N2—C6	168.7 (2)	C18—N5—C24—S2	2.2 (4)
C25—Cd1—N2—C6	−2.9 (16)	C23—S2—C24—N5	−1.5 (3)
C26^i^—Cd1—N2—C6	141.7 (2)	C23—S2—C24—N6	179.2 (3)
O3^i^—Cd1—N3—C17	0.5 (4)	Cd1—O2—C25—O1	1.4 (3)
O1—Cd1—N3—C17	86.9 (3)	Cd1—O2—C25—C21	−179.1 (3)
N1—Cd1—N3—C17	−137.7 (3)	Cd1—O1—C25—O2	−1.5 (3)
O2—Cd1—N3—C17	137.0 (3)	Cd1—O1—C25—C21	179.1 (3)
O4^i^—Cd1—N3—C17	2.3 (3)	C20—C21—C25—O2	11.1 (5)
N2—Cd1—N3—C17	−72.7 (3)	C22—C21—C25—O2	−169.0 (3)
C25—Cd1—N3—C17	111.0 (3)	C20—C21—C25—O1	−169.4 (3)
C26^i^—Cd1—N3—C17	1.6 (3)	C22—C21—C25—O1	10.4 (5)
O3^i^—Cd1—N3—C11	175.6 (2)	C20—C21—C25—Cd1	−144 (8)
O1—Cd1—N3—C11	−97.9 (2)	C22—C21—C25—Cd1	36 (8)
N1—Cd1—N3—C11	37.5 (2)	N3—Cd1—C25—O2	116.9 (2)
O2—Cd1—N3—C11	−47.8 (2)	O3^i^—Cd1—C25—O2	−88.2 (2)
O4^i^—Cd1—N3—C11	177.4 (2)	O1—Cd1—C25—O2	178.6 (3)
N2—Cd1—N3—C11	102.4 (2)	N1—Cd1—C25—O2	11.6 (2)
C25—Cd1—N3—C11	−73.8 (2)	O4^i^—Cd1—C25—O2	−141.69 (19)
C26^i^—Cd1—N3—C11	176.8 (2)	N2—Cd1—C25—O2	29.8 (16)
C5—N1—C1—C2	1.7 (5)	C26^i^—Cd1—C25—O2	−114.5 (2)
Cd1—N1—C1—C2	−167.2 (3)	N3—Cd1—C25—O1	−61.8 (2)
N1—C1—C2—C3	−0.4 (5)	O3^i^—Cd1—C25—O1	93.2 (2)
C1—C2—C3—C4	−1.3 (5)	N1—Cd1—C25—O1	−167.02 (19)
C2—C3—C4—C5	1.8 (5)	O2—Cd1—C25—O1	−178.6 (3)
C1—N1—C5—C4	−1.2 (5)	O4^i^—Cd1—C25—O1	39.7 (2)
Cd1—N1—C5—C4	168.0 (2)	N2—Cd1—C25—O1	−148.9 (14)
C1—N1—C5—C6	−177.1 (3)	C26^i^—Cd1—C25—O1	66.9 (2)
Cd1—N1—C5—C6	−8.0 (4)	N3—Cd1—C25—C21	−88 (8)
C3—C4—C5—N1	−0.5 (5)	O3^i^—Cd1—C25—C21	67 (8)
C3—C4—C5—C6	175.2 (3)	O1—Cd1—C25—C21	−26 (8)
C10—N2—C6—C7	−3.6 (5)	N1—Cd1—C25—C21	167 (8)
Cd1—N2—C6—C7	156.1 (3)	O2—Cd1—C25—C21	155 (8)
C10—N2—C6—C5	174.5 (3)	O4^i^—Cd1—C25—C21	14 (8)
Cd1—N2—C6—C5	−25.7 (4)	N2—Cd1—C25—C21	−175 (7)
N1—C5—C6—N2	23.0 (4)	C26^i^—Cd1—C25—C21	41 (8)
C4—C5—C6—N2	−153.0 (3)	Cd1^ii^—O4—C26—O3	−0.3 (3)
N1—C5—C6—C7	−158.8 (3)	Cd1^ii^—O4—C26—C14	−179.3 (2)
C4—C5—C6—C7	25.2 (5)	Cd1^ii^—O3—C26—O4	0.3 (3)
N2—C6—C7—C8	2.2 (6)	Cd1^ii^—O3—C26—C14	179.3 (2)
C5—C6—C7—C8	−175.8 (3)	C13—C14—C26—O4	178.0 (3)
C6—C7—C8—C9	1.1 (6)	C15—C14—C26—O4	−1.6 (4)
C7—C8—C9—C10	−2.8 (6)	C13—C14—C26—O3	−1.0 (4)
C6—N2—C10—C9	1.7 (5)	C15—C14—C26—O3	179.4 (3)
Cd1—N2—C10—C9	−156.1 (3)	C13—C14—C26—Cd1^ii^	25 (9)
C8—C9—C10—N2	1.5 (6)	C15—C14—C26—Cd1^ii^	−155 (9)

Symmetry codes: (i) *x*+1, *y*, *z*; (ii) *x*−1, *y*, *z*.

### Hydrogen-bond geometry (Å, º)

**Table:**

*D*—H···*A*	*D*—H	H···*A*	*D*···*A*	*D*—H···*A*
N6—H6*A*···O3^iii^	0.88	2.10	2.917 (4)	155
N6—H6*B*···O3^iv^	0.88	2.12	2.996 (4)	173
N4—H4*A*···O4^i^	0.88	2.25	3.101 (4)	162
N4—H4*B*···N5^v^	0.88	2.21	3.066 (4)	163

Symmetry codes: (i) *x*+1, *y*, *z*; (iii) −*x*, −*y*+2, −*z*−1; (iv) *x*+1, *y*, *z*−1; (v) *x*, *y*−1, *z*+1.
